# Machine Learning Classification of Mild Traumatic Brain Injury Using Whole-Brain Functional Activity: A Radiomics Analysis

**DOI:** 10.1155/2021/3015238

**Published:** 2021-11-18

**Authors:** Xiaoping Luo, Dezhao Lin, Shengwei Xia, Dongyu Wang, Xinmang Weng, Wenming Huang, Hongda Ye

**Affiliations:** ^1^Department of Radiology, Wenzhou Chinese Medicine Hospital, Wenzhou, 325000 Zhejiang, China; ^2^Department of Emergency, Wenzhou Chinese Medicine Hospital, Wenzhou, 325000 Zhejiang, China

## Abstract

**Objectives:**

To investigate the classification performance of support vector machine in mild traumatic brain injury (mTBI) from normal controls.

**Methods:**

Twenty-four mTBI patients (15 males and 9 females; mean age, 38.88 ± 13.33 years) and 24 age and sex-matched normal controls (13 males and 11 females; mean age, 40.46 ± 11.4 years) underwent resting-state functional MRI examination. Seven imaging parameters, including amplitude of low-frequency fluctuation (ALFF), fractional amplitude of low-frequency fluctuation (fALFF), regional homogeneity (ReHo), degree centrality (DC), voxel-mirrored homotopic connectivity (VMHC), long-range functional connectivity density (FCD), and short-range FCD, were entered into the classification model to distinguish the mTBI from normal controls.

**Results:**

The ability for any single imaging parameters to distinguish the two groups is lower than multiparameter combinations. The combination of ALFF, fALFF, DC, VMHC, and short-range FCD showed the best classification performance for distinguishing the two groups with optimal AUC value of 0.778, accuracy rate of 81.11%, sensitivity of 88%, and specificity of 75%. The brain regions with the highest contributions to this classification mainly include bilateral cerebellum, left orbitofrontal cortex, left cuneus, left temporal pole, right inferior occipital cortex, bilateral parietal lobe, and left supplementary motor area.

**Conclusions:**

Multiparameter combinations could improve the classification performance of mTBI from normal controls by using the brain regions associated with emotion and cognition.

## 1. Introduction

Traumatic brain injury (TBI), a major public health problem and a leading cause of disability, affects half the world's population [1]. Approximately 70%-90% of TBI patients are mild TBI (mTBI), and 30-40% of whom cannot fully recover even at 6 months postinjury [1, 2]. Patients with mild head injury often manifest as dizziness, headache, and memory and attention deficit, which was considered to be associated with abnormal changes of brain networks [3]. Recently, functional and structural neuroimaging methods have been widely used to address the functional and morphological changes of mTBI [4–11]. Zhou et al. found abnormal functional connectivity within the default mode network in mTBI patients, which was associated with cognitive neurological dysfunction and posttraumatic symptoms (i.e., depression, anxiety, fatigue, and postconcussion syndrome) [12]. Nakamura et al. found that mTBI was associated with changes in the “small world” networks [13]. Zhan et al. found decreased ReHo value in the left insula, left pre-/postcentral gyrus, and left supramarginal gyrus in mTBI patients [14]. However, the potential neurobiological mechanism of the mTBI left unclear.

Most current studies focus attentions on investigating group differences between two different labels (knowing the classes of all subject before statistics); however, group-based methods cannot classify different types for individual classification and are not sensitive for feature selection [15]. Support vector machine (SVM) classifier is an efficient and sensitive neuroimaging biological indicator for feature selection and classification. There is a growing application of the SVM algorithm into several diseases, such as insomnia [16, 17], epilepsy [15], and autistic spectrum disorder [18]. However, the mTBI has not been studied. Differences in brain regions in mTBI were not the same when we analyzed the between-group differences by different neuroimaging methods, which may be associated with the sensitivity of different methods in searching features (brain areas). Therefore, we hypothesized that the combination of different neuroimaging methods may improve the sensitivity for feature selection. To address these hypotheses, the present study is the first to apply the SVM algorithm to perform the classification for mTBI.

## 2. Materials and Methods

### 2.1. Subjects

This case-control study comprised 170 subjects from our hospital between May 2014 and May 2021, among whom a total of 146 subjects were excluded, including 139 subjects unmatched diagnosis with mTBI, 4 mTBI with more than 1.5 mm maximum translation in *x*, *y*, or *z* directions and/or 1.5 degree of motion rotation, and 3 mTBI with missing data. Finally, 24 patients with acute mTBI (15 males and 9 females; mean age, 38.88 ± 13.33 years; mean years of education, 8.88 ± 3.58 years; and mean time of postinjury, 3.58 ± 3.28 days) and 24 age and sex-matched (13 males and 11 females; mean age, 40.46 ± 11.4 years; and mean years of education, 8.54 ± 3.41 years) healthy controls were included. All subjects were asked to complete the following questionnaires, including the Glasgow Coma Scale (GCS), Disability Rating Scale (DRS), Motor Assessment Scale (MAS), Agitated Behavior Scale (ABS), Hamilton Anxiety Scale (HAMA), Clinical Dementia Rating (CDR), Mini Mental State Examination (MMSE), Activates of Daily Living (ADL), and Beck Depression Inventory (BDI).

Inclusion criteria for patients with acute mTBI were as follows: (a) have a diagnosis of mTBI within two weeks, (b) age between 18 and 65 years, (c) time of lack of consciousness less than 30 min, and (d) time of posttraumatic amnesia less than 24 hours. Exclusion criteria for patients with acute mTBI were as follows: (a) involvement in litigation, (b) a history of psychiatric disorders, (c) a history of addiction, and (d) a history of traumatic brain injury. This study was approved by the Human Research Ethics Committee in accordance with the Declaration of Helsinki, and written informed consent was obtained.

### 2.2. MRI Parameters

MRI data were acquired with a clinical 3-Tesla MRI scanner (Trio Tim, SIEMENS, Erlangen, Germany), including T1WI, T2WI, T2-FLAIR, high-resolution T1WI, functional MRI, and SWI. A total of 176 three-dimensional high-resolution anatomical T1-weighted volumes were acquired in a sagittal orientation (rapid-gradient-echo sequence, repetition time = 1900 ms, echo time = 2.26 ms, thickness = 1.0 mm, matrix = 256 × 256, and field of view = 240 mm × 240 mm). For functional images, a total of 250 volumes (Echo-Planar Imaging pulse sequence, 30 transverse slices, repetition time = 2000 ms, echo time = 40 ms, thickness = 4.0 mm, matrix = 64 × 64, field of view = 240 mm × 240 mm, and flip angle 90°) were acquired.

### 2.3. Data Processing

All functional MRI data preprocessing were performed with DPABI (version 2.1, http://rfmri.org/DPABI) toolbox. First, the first ten volumes were deleted, and the remaining volumes were converted their data format. The following steps of slice timing, head motion correction, spatial normalization, smooth (Gaussian kernel of 8 × 8 × 8 mm^3^), linear regression of possible spurious covariates, linearly detrended, and temporally band-pass filtered (0.01-0.1 Hz) were performed for data preprocessing. After the step of head motion correction, a “head motion scrubbing regressors” procedure was implemented, and the subjects who had more than 1.5 degree of motion rotation and/or 1.5 mm maximum translation in *x*, *y*, or *z* directions were excluded. Furthermore, the head motion effect was regressed out with Friston 24 head motion parameter model. During the step of spatial normalization, all data were spatially normalized to Montreal Neurological Institute (MNI) space and resampled at a resolution of 3 × 3 × 3 mm^3^.

### 2.4. Feature Selection and Binary Classification

We calculated seven MRI parameters, including ALFF, fALFF, ReHo, degree centrality, long-term FCD, short-term FCD, and VMHC. The maps of MRI parameters were segmented into 116 regions of interest (ROIs) using the automated anatomical labeling (AAL) atlas. The total of 812 features was extracted in the following classification with multivariate pattern analysis (MVPA).

We used a LIBSVM toolbox (http://www.csie.ntu.edu.tw/~cjlin/libsvm/) to perform the classification, and a 5-fold cross-validation was used to validate the classification performance of the classifier. Permutation test was used to evaluate the probability of the classification performance for 5000 times randomly. The clusters of brain regions with higher than 70% of classification accuracy were considered as accuracies. The area under curve (AUC), sensitivity, and specificity of the classifier were quantified.

### 2.5. Statistical Analyses

Comparisons of demographic factors were performed using two-sample *t*-tests. Chi-square (*χ*^2^) test was used for categorical data. Statistical analysis was performed using IBM SPSS 21.0 version. Data are presented as mean ± standard deviation. All the quoted results are two-tailed values, and *p* < 0.05 was considered as statistically significant.

## 3. Results

### 3.1. Sample Characteristics

There were no significant differences in mean age (*t* = −0.442, *p* = 0.66), sex (*χ*^2^ = 0.343, *p* = 0.558), and educational level (*t* = 0.33, *p* = 0.743) between the healthy controls and patients with mTBI. Compared with healthy controls, patients with mTBI had higher HAMA score (*t* = 5.077, *p* < 0.001), ADL score (*t* = 4.654, *p* < 0.001), and BDI score (*t* = 3.808, *p* = 0.001), and a lower MMSE score (*t* = −2.284, *p* = 0.03). The mean time between injury and MRI examination of patients with mTBI was 3.58 ± 3.28 days. The mean GCS score, DRS score, MAS score, and ABS score in patients with mTBI were 14.42 ± 0.88, 2.58 ± 2.36, 44.38 ± 5.86, and 14.42 ± 0.78, respectively. The details are shown in Table 1.

### 3.2. Classification Performance

**Figure 1:**
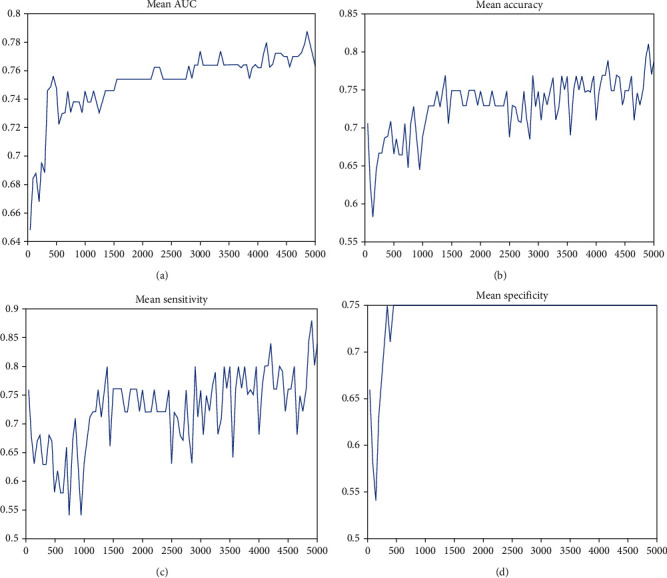
Schematic diagram overview of machine learning classification framework. Note: this figure shows the classification of the combination of ALFF, fALFF, DC, VMHC, and short-term FCD in distinguishing the mTBI from the normal controls. The classification received the highest (a) AUC value, (b) classification accuracy, (c) sensitivity, and (d) specificity among all combination.

First, we compared the classification performances of the seven MRI parameters and found they could not differentiate well between healthy controls and patients with mTBI (AUC: 0.66 ± 0.03, range, 0.61~0.69; accuracy rate: 66.4% ± 3.4%, range, 60.2%~70.9%; sensitivity: 64.1% ± 7.9%, range, 49.0%~75.0%; and specificity: 68.4% ± 5.6%, range, 61.0%~75.0%). Second, we combined these MRI parameters and found the features with the highest contributions to the classification to discriminate between mTBI and healthy controls. We found that the combination with ALFF, fALFF, DC, VMHC, and short-term FCD significantly reached up the classification accuracy, sensitivity, and specificity and received the highest classification performances among all combination with classification accuracy of 81.1% (*p* < 0.001), sensitivity of 88.0% (*p* < 0.001), and specificity of 75.0% (*p* < 0.001) (Figure 1).

### 3.3. Consensus Features and Region Weight

In this study, all consensus features were mapped to AAL116 template (116 brain regions), and each of the 116 brain regions was given a weight value which indicates the contribution to classification model. For the combination with ALFF, fALFF, DC, VMHC, and short-term FCD, Table 2 shows the weight ranking of the 116 brain regions from highest to lowest.

Among the 116 brain regions, a total of 51 brain regions showed higher contributions to the classification than the average weight value (contribution), including the bilateral cerebellum, left orbitofrontal cortex, left cuneus, left temporal pole, right inferior occipital gyrus, bilateral parietal lobe, and left supplementary motor area (Table 2).

## 4. Discussion

In this case-control study, we documented two novel findings. First, we developed an SVM classifier that was a useful neuroimaging biomarker for mTBI classification. We found that the combination with ALFF, fALFF, DC, VMHC, and short-term FCD received the highest classification performances among all combination (accuracy = 81.1%, sensitivity = 88.0%, and specificity = 75.0%). Second, the consensus brain regions with the highest contributions to classification were located in the bilateral cerebellum, left orbitofrontal cortex, left cuneus, left temporal pole, right inferior occipital gyrus, bilateral parietal lobe, and left supplementary motor areas (contribution above the average value among 116 brain regions).

Our study is the first to apply the SVM classifier to find a promising model for mTBI classification. Although several previous studies have offered insights into brain functional and structural abnormalities of mTBI using traditional group-level statistical differences based on one single imaging method, they could not be translated into predictive or diagnostic neurobiological biomarkers for mTBI. The emergence of radiomics has broadened the scope of routine medical imaging, which carried multimodality medical information to reflect the development and progression of diseases [19, 20]. Machine learning classification based on the radiomics strategy allows detecting subtle, nonstrictly localized effects that may remain invisible to the conventional analysis with univariate statistics [21, 22], which are being increasingly used in functional MRI data [15, 16]. These findings could explain the high classification performance of the SVM classifier.

Cerebellum is associated with emotion, motor, and advanced cognitive function [23]. The cerebellum anterior lobe is associated with sensorimotor function, and the cerebellum posterior lobe is associated with the regulation of coordinating movement, balance and sleep, and emotional changes [24–28]. Brain volume atrophy and reduction of metabolism functional activity can be found in subjects after TBI [29–31]. Peskind et al. found that soldiers with mTBI showed reduction of glucose metabolism in the cerebellar vermis, cerebellar hemisphere, and pons and functional deficits in attention, language, and working memory [31]. In addition, cerebellar activation was also significantly reduced during auditory-related task stimulation [30]. These studies suggest that the cerebellum plays an important role in the neuropathological basis of mTBI, which supports our findings of high contributions of the cerebellum to the SVM classifier.

The prefrontal lobe is one of the brain areas that are most vulnerable to the mTBI. Even minor brain damage can easily cause a damage of the frontal lobe. Studies have found that abnormal functional changes in the frontal lobe are one of the neural mechanisms of emotional numbness, attention, planning, high alertness, and psychological avoidance in patients with posttraumatic injury [32–34]. Keightley et al. found that adolescents with mTBI showed weaker working memory and language function and reduced brain activity in supplementary motor areas, dorsolateral prefrontal lobe, and superior parietal lobe than that of healthy adolescents [35]. Pardini et al. and Jantzen et al. found that parietal lobe and orbitofrontal cortex are associated with severity of mTBI and postconcussion symptoms [36, 37]. Our findings support these studies. Therefore, the abnormal functional changes in the frontal-parietal lobe may be associated with the posttraumatic injury severity and symptoms, which contribute to the high contributions to the SVM classifier.

Abnormal functional connectivity between temporal pole and parietal lobe and decreased glucose metabolism in these two areas were found in mTBI patients relative to normal controls [31, 38, 39]. The temporal pole is closely related to the functions such as social interaction, face recognition, semantic memory, mental speculation, and emotion and is responsible for the synthesis of complex and finely processed perceptual input of internal emotions [40]. The abnormal function of the temporal pole in mTBI patients will help us understand the biological mechanism of daily life disorders of mTBI.

## 5. Conclusions

In this study, we developed an SVM classifier that can be severed as a promising sensitive neuroimaging biomarker for mTBI classification based on a combination of multiple imaging indicators. Our analysis using the model showed that the bilateral cerebellum, left orbitofrontal cortex, left cuneus, left temporal pole, right inferior occipital gyrus, bilateral parietal lobe, and left supplementary motor areas exhibited the highest contributions to the classification model. These findings may expand our understanding of the neurobiological mechanism of mTBI. However, there are several limitations that should be addressed. First, the sample size of our study was relatively small. A larger number of sample sizes and multiple center studies are necessary to corroborate our findings. Second, the data of subacute mTBI and follow-up were scarce. Third, this study only used SVM to perform the classification, and other classification methods should be introduced to compare their performances. Fourth, location and size of the lesion, disease of severity, and subtype of mild traumatic brain injury were not considered in the classification.

## Figures and Tables

**Table 1:** Demographic and clinical features of patients with acute mTBI and healthy controls.

	mTBI	Healthy controls	*t* value	*p* value
Age, years	38.88 ± 13.33	40.46 ± 11.40	-0.442	0.66
Sex (male, female)	24 (15, 9)	24 (13, 11)	0.343	0.558
Education, years	8.88 ± 3.58	8.54 ± 3.41	0.330	0.743
Postinjury, days	3.58 ± 3.28	N/A	N/A	N/A
GCS	14.42 ± 0.88	N/A	N/A	N/A
DRS	2.58 ± 2.36	N/A	N/A	N/A
MAS	44.38 ± 5.86	N/A	N/A	N/A
ABS	14.42 ± 0.78	N/A	N/A	N/A
HAMA	3.83 ± 3.61	0.08 ± 0.28	5.077	<0.001
MMSE	29.04 ± 1.63	29.83 ± 0.20	-2.284	0.03
ADL	21.71 ± 8.07	14.04 ± 6.06	4.654	<0.001
BDI	1.58 ± 1.91	0.08 ± 0.28	3.808	0.001

**Table 2:** Weight ranking of the 116 brain regions to the classification of the combination with ALFF, fALFF, DC, VMHC, and short-term FCD.

	ROI weight	Voxel size
Vermis_10	1.948	34
Cerebellum_9_R	1.470	156
Cerebellum_10_L	1.465	40
Cerebellum_9_L	1.455	158
Frontal_Mid_Orb_L	1.417	224
Frontal_Sup_Orb_L	1.398	280
Cuneus_L	1.353	472
Cerebellum_Crus2_R	1.329	539
Cerebellum_7b_L	1.265	98
Cerebellum_Crus2_L	1.257	543
Frontal_Mid_Orb_L	1.256	273
Temporal_pole_Mid_L	1.222	177
Occipital_Inf_R	1.220	316
Parietal_Sup_L	1.136	575
Paracentral_lobule_R	1.134	221
Frontal_Sup_R	1.118	1120
Cuneus_R	1.117	416
Cerebellum_Crus1_R	1.103	723
Occipital_Inf_L	1.103	263
Vermis_7	1.102	54
Calcarine_L	1.076	649
Occipital_Sup_R	1.074	407
Rectus_L	1.070	258
Postcentral_R	1.055	1050
Paracentral_lobule_L	1.053	340
Precentral_R	1.009	941
Parietal_Inf_R	1.008	397
Occipital_Sup_L	0.998	373
Cerebellum_10_R	0.997	37
Cerebellum_7b_R	0.975	78
Cerebellum_8_L	0.961	303
Cerebellum_6_L	0.960	524
Vermis_9	0.959	50
Temporal_Inf_R	0.959	1076
Occipital_Mid_L	0.954	947
Cerebellum_Crus1_L	0.950	725
Lingual_L	0.945	662
Supp_motor_area_L	0.937	630
Frontal_Mid_R	0.931	1448
Calcarine_R	0.925	528
Temporal_Mid_L	0.922	1437
Parietal_Sup_R	0.921	569
Cerebellum_4_5_L	0.919	352
Frontal_Sup_medial_L	0.918	847
Lingual_R	0.916	683
Angular_R	0.916	511
Temporal_pole_Sup_R	0.918	325
Cerebellum_6_R	0.908	532
Precuneus_R	0.908	927
Temporal_Sup_R	0.907	942
Frontal_Sup_L	0.890	987
Angular_L	0.860	341
Precuneus_L	0.851	1008
Cingulum_post_L	0.850	111
Frontal_Inf_Tri_L	0.848	675
Frontal_Mid_L	0.847	1323
Temporal_pole_Sup_L	0.828	329
Temporal_Sup_L	0.825	694
Temporal_pole_Mid_R	0.822	264
Cerebellum_8_R	0.810	298
Cerebellum_3_R	0.801	65
Occipital_Mid_R	0.796	578
Supp_motor_area_R	0.790	695
Vermis_4_5	0.788	176
Frontal_Sup_medial_R	0.787	589
Frontal_Inf_Tri_R	0.783	560
Supramarginal_R	0.779	562
Precentral_L	0.764	931
Heschl_R	0.763	60
Frontal_Mid_Orb_R	0.762	296
Frontal_Sup_Orb_R	0.752	296
Frontal_Inf_Orb_L	0.752	504
Cerebellum_3_L	0.750	42
Supramarginal_L	0.750	357
Fusiform_L	0.747	665
Temporal_Inf_L	0.745	948
Vermis_1_2	0.738	9
Rectus_R	0.728	208
Parietal_Inf_L	0.723	687
Cerebellum_4_5_R	0.719	239
Frontal_Inf_Oper_R	0.715	396
Caudate_L	0.703	270
Postcentral_L	0.702	1069
Fusiform_R	0.688	759
Pallidum_L	0.685	76
Vermis_6	0.675	87
Amygdala_L	0.672	63
Putamen_L	0.660	280
Frontal_Inf_Orb_R	0.660	498
Frontal_Mid_Orb_R	0.657	271
Vermis_8	0.652	60
Insula_R	0.646	497
Rolandic_Oper_L	0.645	301
Cingulum_Mid_L	0.635	579
Olfactory_L	0.632	80
Thalamus_R	0.631	296
Frontal_Inf_Oper_L	0.628	309
Parahippocampal_L	0.624	298
Pallidum_R	0.621	67
Cingulum_Ant_R	0.621	385
Temporal_Mid_R	0.619	1311
Cingulum_Ant_L	0.598	425
Thalamus_L	0.596	280
Cingulum_Mid_R	0.574	612
Vermis_3	0.549	62
Rolandic_Oper_R	0.548	404
Heschl_L	0.548	72
Olfactory_R	0.538	88
Cingulum_post_R	0.522	69
Caudate_R	0.511	287
Parahippocampal_R	0.506	318
Hippocampus_L	0.493	279
Amygdala_R	0.474	73
Insula_L	0.465	545
Putamen_R	0.436	309
Hippocampus_R	0.411	282

## Data Availability

The data that support the findings of this study are available from the corresponding author upon reasonable request.
